# German National Proficiency Scales in Biology: Internal Structure, Relations to General Cognitive Abilities and Verbal Skills

**DOI:** 10.1002/sce.21227

**Published:** 2016-04-28

**Authors:** NELE KAMPA, OLAF KÖLLER

**Affiliations:** ^1^ Leibniz Institute for Science and Mathematics Education (IPN)Christian‐Albrechts‐UniversitätD‐24118KielGermany

## Abstract

National and international large‐scale assessments (LSA) have a major impact on educational systems, which raises fundamental questions about the validity of the measures regarding their internal structure and their relations to relevant covariates. Given its importance, research on the validity of instruments specifically developed for LSA is still sparse, especially in science and its subdomains biology, chemistry, and physics. However, policy decisions for the improvement of educational quality based on LSA can only be helpful if valid information on students’ achievement levels is provided. In the present study, the nature of the measurement instruments based on the German Educational Standards in Biology is examined. On the basis of data from 3,165 students in Grade 10, we present dimensional analyses and report the relationship between different subdimensions of biology literacy and cognitive covariates such as general cognitive abilities and verbal skills. A theory‐driven two‐dimensional model fitted the data best. Content knowledge and scientific inquiry, two subdimensions of biology literacy, are highly correlated and show differential correlational patterns to the covariates. We argue that the underlying structure of biology should be incorporated into curricula, teacher training and future assessments.

## INTRODUCTION

National and international large‐scale assessments (LSA) have a major impact on educational systems. They have influenced political stakeholders’ decision making over the past decades and will do so in the future (for Germany, see Neumann, Fischer, & Kauertz, 2010). This development raises fundamental questions about the validity of the implemented LSA measures. Since educational reports (e.g., National Assessment for Educational Progress [NAEP] nation's report card) form an important basis for educational policy to improve the quality of teaching and learning, the measures for the reported abilities should be thoroughly validated. Surprisingly and given their importance, research on the validity of instruments for LSA is still sparse. This is especially true in science and its subdomains biology, chemistry, and physics.

To cover abilities as comprehensive and efficient as possible, both the assessment and the reporting are often multidimensional (e.g., Martin, Mullis, Foy, & Stanco, 2012; Organisation for Economic Co‐operation and Development [OECD], 2007). The definitions and theoretical frameworks of scientific literacy of national and international LSA usually cover multiple aspects of the construct to be measured (e.g., Bybee, 1997; Driscoll, Avallone, Orr, & Crovo, 2010; OECD, 2013; Shavelson et al., 2008). Based on these conceptualized subdimensions of scientific literacy, validity studies have to justify the theoretical considerations with respect to the internal structure in terms of multidimensionality of the measures.

Validity aspects are also expressed in the relations of the subdimensions to relevant covariates. Students refer to a variety of abilities to solve typical scientific problems encountered in LSA. Two of the most important abilities that sometimes are also criticized as being confounding variables are general cognitive abilities and verbal skills (Gustafsson & Balke, 1993; Taboada & Rotherford, 2011). On the one hand, general cognitive abilities are necessary to solve different scientific problems from interpreting a table to setting up an experiment (Gustafsson & Balke, 1993). On the other hand, reading is a pivotal part of science learning (Ford, Brickhouse, Lottero‐Perdue, & Kittleson, 2006), because a major part of science in school is transmitted through written documents.

Since teaching and teacher training in science are subject specific in Germany, we concentrate in this paper on biology. We chose biology because it starts early on in German secondary schools (usually in Grade 5) is the most popular subdiscipline in science and is taught in all school types of secondary education. More precisely, we investigate validity aspects of an item pool which is used in the German national standard–based assessments of students’ abilities in biology at the end of lower secondary school. We explore its internal structure according to the postulated dimensionality and its relationship to external constructs, i.e., verbal skills and general cognitive abilities.

## THEORETICAL BACKGROUND

To frame our study on the German Educational Standards in Biology, we need to incorporate several perspectives of scientific and biology literacy. We first draw upon conceptualizations of scientific and biology literacy as well as its assessment. By synthesizing both views, we will split up biology literacy into a scientific inquiry and a content dimension.

### Structure of Scientific and Biology Literacy

A literature review on the internal structure of scientific literacy reveals several conceptualizations: According to Bybee (1997), the term scientific literacy describes all abilities an individual needs to solve scientific problems in real‐world situations. Because scientific literacy is a fuzzy concept, its frameworks (e.g., Bybee, 1997; Fives, Hubner, Birnbaum, & Nicolich, 2014; Gott, Duggan, & Johnson, 1999; Gräber, Nentwig, & Nicolson, 2002; Harlen, 2001; Hodson, 1992; Osborne, 2007; Shavelson et al., 2008; Westby & Torres‐Velásquez, 2000) are quite diverse. The majority of the frameworks describe content/conceptual aspects and at least one process aspect (see Table 1). The most popular framework was introduced by Bybee (1997) and formed the basis for the first conceptualization of the Programme for International Student Assessment (PISA). In this framework, different levels are described, namely (a) nominal scientific literacy, (b) functional scientific literacy, (c) conceptual and procedural scientific literacy, as well as (d) multidimensional scientific literacy. Another influential framework was established by Shavelson et al. (2008) who differentiated between the following knowledge types and corresponding reasoning within science achievement: (a) declarative, (b) procedural, (c) schematic, and (d) strategic knowledge and reasoning. Declarative knowledge and reasoning relate to the knowledge of facts and concepts. Procedural knowledge and reasoning describe step‐by‐step sequences of procedures as well as condition action. Schematic knowledge and reasoning connect the declarative and procedural knowledge when individuals create and use mental models. The strategic knowledge and reasoning describes audience‐ and situation‐appropriate applications of the knowledge types and corresponding reasoning. As Shavelson et al. (2008) aptly stated, declarative knowledge refers to identifying science principles and procedural knowledge refers to using scientific inquiry. Declarative and procedural knowledge serve as the basis for the Nation's Report Cards of the NAEP (Driscoll et al., 2010). The same two subdimensions have not only been stated for science ability but also theoretically hypothesized for biology literacy. However, different authors labeled the subdimensions somewhat differently and defined additional subfacets within the subdimensions. For example, on the one hand, the sub‐dimension procedural understanding in biology (which is nowadays called concepts of evidence; see http://community.dur.ac.uk/rosalyn.roberts/Evidence/cofev.htm) in the framework of Roberts and Gott (1999; see also, Gott et al., 1999) explicitly includes variable identification, underlying and nonlinear relationships as well as representative sampling. On the other hand, the subdimension science practices of the NAEP framework (Winick, Avallone, Smith, & Crovo, 2008) includes identifying and using science principles as well as using scientific inquiry and technological designs.

**Table 1:** Synopsis of the Dimensions of Scientific and Biology Literacy From Example Definitions as well as Assessment and Standard Frameworks

Framework	Content Dimension	Process Dimension	Additional Dimensions
**Bybee (1997)**	Nominal scientific literacy Functional scientific literacy Conceptual scientific literacy	Procedural scientific literacy	Multidimensional scientific literacy
Fives et al, (2014)	Role of science	Role of scienceScientific thinking and doing	Science and society Science media literacy Mathematics in science Science motivation and beliefs
Gott et al. (1999)	Conceptual understanding/facts	Procedural understanding/skills	
Gräber et al. (2002)	Subjective competence	Procedural competence	Epistemological competence
			Ethical competence Learning competence Social competence Communicative competence
Harlen (2001)	Scientific concepts	Scientific processes	Areas of application Situations
Hodson (1992)	Scientific knowledge	Scientific method	
Osborne (2007)	Conceptual	Cognitive	Ideas‐About‐Science
			Social and Affective
**Shavelson et al. (2008)**	Declarative knowledge and reasoning	Procedural knowledge and reasoning	Strategic knowledge and reasoning Schematic knowledge and reasoning
Westby and Torres‐Velasquez (2000)	Knowing science	Doing science	Talking science Scientific habits of minds
**Driscoll et al. (2010)**	Science content	Science practices	
**PISA (2013) competencies**	Content knowledge	Procedural knowledge	Epistemic knowledge
**PISA (2013) knowledge types**	Explain phenomena scientifically	Evaluate and design scientific enquiry Interpret data and evidence scientifically	
**NEPS (2013)**	Knowledge of science	Knowledge about science	
**TIMSS (2012) domains**	Content domains	Cognitive domains	
**German Educational Standards (2005)**	Content knowledge	Scientific Inquiry	Assessment Communication
**US Educational Standards (2008)**	Science content	Science practices	
**GCSE exam definitions**	Subject content	Controlled assessment	

*Note*. PISA = Programme for International Student Assessment, NEPS = National Educational Panel Study, GCSE = General Certificate of Secondary Education, TIMSS = Trends in Mathematics and Science Study.

Even though the process aspects of science are conceptualized differently in the listed frameworks (e.g., focus on practical work or on philosophical issues), we will subsequently address the two dimensions of scientific/biology literacy as content knowledge and scientific inquiry. This simplification is necessary when comparing different frameworks for different purposes since the different levels of application of the frameworks lead to different foci and broadness of the dimensions. Once we pursue with this simplification, every framework incorporates a content/conceptual and a scientific process dimension. A subset of the frameworks also includes either epistemological beliefs (e.g., Osborne, 2007) or communicative (e.g., Westby & Torres‐Velásquez, 2000) dimensions. Even though both dimensions are essential to describing scientific literacy, we can only assume that until now, no consensus about these dimensions of scientific literacy is reached. We will thus focus on the two consensual dimensions content knowledge and scientific inquiry. By content knowledge we do not refer to knowledge recall—just like any other framework of scientific literacy—but rather to solving problems by actively working and dealing with scientific content (Pant et al., 2012).

To further understand the conceptualization of scientific literacy, several researchers have investigated the different aspects within scientific inquiry in more detail. For example, Chinn and Malhotra (2002) identified three cognitive processes (developing theories, designing studies, and generating research questions) and three epistemological aspects (social construction of knowledge, purpose of research, and theory‐ladenness of methods) of scientific inquiry that guide the research process. A German research group conceptualized the four aspects: (a) formulating questions, (b) generating hypotheses, (c) planning of investigation, and (d) interpreting data (Kremer, Specht, Urhahne, & Mayer, 2014). In our framework, scientific inquiry incorporates scientific investigation—which includes the four mentioned aspects—scientific modeling as well as scientific theorizing, with the latter two looking at science more abstractly (Pant et al., 2012). Scientific modeling deals with the functions of models, their uses and limitations. Scientific theorizing deals with the characteristics and the development of science.

Some of the frameworks described above provide the theoretical basis of LSA and educational monitoring (see frameworks in bold in Table 1). The aspects of content knowledge and scientific inquiry can be found in (a) educational standards, (b) subject‐specific curricula, (c) frameworks of scientific literacy, and (d) national and international LSA. Comprehensive empirical investigations testing the dimensionality of scientific literary in general and biology literacy in specific for LSA are rare.

### Standards and Assessment in Science and Biology Education in Germany

In most countries, the assessment of scientific and biology literacy targets multiple subdimensions. For example, in NAEP the underlying framework distinguishes between a content dimension and practical dimensions of scientific literacy (Driscoll et al., 2010). The framework of PISA (OECD, 2013) and the German National Educational Panel Study (NEPS; Hahn et al., 2013) both cover knowledge of science and knowledge about science with the latter comprising process aspects. The German Educational Standards provide the theoretical framework for the national assessment in biology, chemistry, and physics (The Standing Conference of the Ministers of Education and Cultural Affairs of the Länder in the Federal Republic of Germany [KMK], 2005a, 2005b, 2005c). In Germany, science is taught as an integrated subject in primary school (Grades 1–4). In secondary school (Grades 5–12); however, it is mostly taught in three different subjects—biology, chemistry, and physics. The standards for all three domains are split into one subject‐specific component—content knowledge—and three process dimensions: (a) scientific inquiry, (b) scientific judgment and evaluation, as well as (c) communication. Such a distinction between one content dimension and several process dimensions can also be found in other countries (see Table 1). In the United States, for instance, the National Standards are represented by the categories (a) science as inquiry, (b) history of science, (c) nature of science, and (d) the designed world (Winick et al., 2008).

These examples underline that educational assessment usually covers multidimensional constructs (The Assessment and Qualifications Alliance [AQA], 2011; Educational Excellence, 2012; Winick et al., 2008). However, the underlying politically motivated standards are not always derived from theory or empirical findings. Instead, the procedure is normative in nature. Usually, political stakeholders together with educational researchers and didactics experts decide a priori on relevant dimensions of student achievement. In Germany, this led to Educational Standards for the four content areas content knowledge, scientific inquiry, evaluation, and communication. Supporting empirical educational research as a backup for the politically intended abilities structure of LSA is often missing. Therefore, research tackling the dimensionality of scientific literacy in standard‐based LSA and its relationship to other abilities or skills is needed. Exploring the dimensional structure of these assessments helps interpreting the results in a meaningful way since it shows at what fine‐grained level the results should be reported. The reporting of the results should always be justified by theoretical rationale and empirical evidence. Our overview of frameworks of scientific literacy (see Table 1) already justifies the reporting of LSA results for the two dimensions content knowledge and scientific inquiry. The next step will be to examine whether the data provided for the German Educational Standards can empirically be used for that purpose. We argue that such research issues should constitute the basis of any improvements or amendments made to the original considerations of LSA.

### Research on the Relation Between Content Knowledge and Scientific Inquiry in Scientific Literacy

The major focus of PISA 2006 was on scientific literacy (OECD, 2009). Results were reported for three dimensions of scientific literacy: (a) identifying scientific issues, (b) explaining phenomena scientifically, and (c) using scientific evidence with the former two more closely related to content knowledge and the latter one to scientific inquiry. The correlations between these three dimensions, however, were quite high with values between *r* = .89 and .93 (after correcting for measurement error). Based on the national data of the German PISA 2003 study, which was expanded by items on specific cognitive processes, structural equation models (SEM), showed a more fine‐grained and complex picture (Senkbeil, Rost, Carstensen, & Walter, 2005). Analyses revealed seven cognitive subdimensions that were labeled as follows: (a) convergent thinking, (b) divergent thinking, (c) dealing with graphs, (d) dealing with mental models, (e) dealing with numbers, (f) verbalize facts, and (g) evaluating, with (f) more closely related to content knowledge, whereas (c) and (d) describe cognitive demands that can be ascribed to scientific inquiry. These cognitive demands formed distinguishable latent factors with correlations between *r* = .56 and .77.

Further studies have shown differential patterns of the two subdimensions for groups of participants. For instance, reanalyses of PISA 2003 field study data showed gender differences between the more curriculum‐oriented national test and the more literacy oriented international test (Rost, Prenzel, Carstensen, Senkbeil, & Groß, 2004). Boys outperformed girls in the subdimensions subject knowledge and constructing mental models. In another study on the impact of the socioeconomic background on scientific literacy, Turmo (2004) demonstrated that abilities in the process dimension were more strongly associated with socioeconomic status than in the product dimension. In summary, previous research suggests that different, although substantially correlated subdimensions of scientific literacy do exist. We first provide empirical evidence for the validity of reporting the results on such a coarse dimensional level. Hitherto, the distinction between content knowledge and scientific inquiry in the domain of biology has not been explicitly investigated.

### Research on the Relation Between Scientific/Biology Literacy and General Cognitive Abilities

General cognitive abilities are the strongest single predictor of interindividual differences in students’ achievement and knowledge acquisition (Gustafsson & Balke, 1993). Different approaches describe what constitutes general cognitive abilities, but most often it is understood as a higher order factor named g (Carroll, 1993; Spearman, 1904). Furthermore, knowledge within a specific domain is seen as an integral part of general cognitive abilities (Rolfhus & Ackerman, 1999). In Germany, there has been a vivid debate on whether the assessment of academic achievement in various domains and the assessment of general cognitive abilities are identical (e.g., Rindermann, 2006). There is evidence that they are different, but overlap substantially (Baumert, Lüdtke, Trautwein, & Brunner, 2009). For instance, a representative longitudinal study in the United Kingdom showed that general cognitive abilities played an important role in educational achievement in 25 domains, among them English, music, and biology (Deary, Strand, Smith, & Fernandes, 2007). More specifically, the general cognitive ability at age 11 and the General Certificate of Secondary Education (GCSE) examinations as a general achievement indicator showed an average (corrected for measurement) correlation of *r* = .81. The correlation to the GCSE examination in biology is *r* = .51. Another cross‐sectional study in the context of NAEP showed a manifest correlation between general cognitive abilities and science achievement as measured by a multiple‐choice science test of *r* = .67 in a sample of 10th‐ and 11th‐grade students (Lau & Roeser, 2002). This was also the strongest predictor among the observed variables (e.g., competence beliefs, classroom management, or extracurricular engagement). In summary, there is ubiquitous and strong evidence that the relationship between general cognitive abilities and academic achievement is high and stable. Regarding the two subdimensions of content knowledge and scientific inquiry, one could assume that general cognitive abilities are stronger related to scientific inquiry than to content knowledge, because it is presumably more influenced by a general ability to plan, think abstractly, and to reason whereas content knowledge might be only moderately related to these general abilities (cf. Bond, 1989).

### Research on the Relation Between Scientific or Biology Literacy and Verbal Skills

Verbal skills shape the academic future of students significantly, and differences in knowledge and vocabulary lead to achievement differences for different populations, particularly in science (Cromley, Snyder‐Hogan, & Luciw‐Dubas, 2010; Greenleaf et al., 2011). Reading skills did not play a major role in the research on science achievement and science education in the past (Greenleaf et al., 2011; Wellington & Osborne, 2001). Science is to a great extent transmitted, learned, and documented through written documentation and through discourse (Brown, Reveles, & Kelly, 2005). Beside the science classrooms, students encounter science also in out‐of‐school contexts by reading magazines and textbooks as well as viewing documentaries rather than from own scientific investigations. Put differently, ideas and theories of science are cultural accomplishments that are in great parts transmitted to students through language (Ford et al., 2006). This leads us to the fact that scientific literacy cannot be acquired without a sufficient ability to read in order to comprehend the language of instruction (Taboada & Rotherford, 2011; Wellington & Osborne, 2001).

Verbal skills are important for science achievement at least for two reasons: First, the language of science is usually an academic language that features other characteristics than narrative texts or the common language used in school classes. According to Westby and Torres‐Velásquez (2000), the language of science is “lexically more dense and structurally more complex” (p. 105). Persons literate in science have to be able to encode and reflect on a specific scientific, formalized language. Second, many standardized achievement tests on scientific literacy are administered as paper‐and‐pencil tests (Greenleaf et al., 2011). This procedure obviously requires students to have the ability to sufficiently read and write to complete the tasks. So in a testing situation, verbal skills can also be seen as a confounding variable. These two reasons exemplify the importance of verbal skills in validity studies as well as for research on its impact on scientific literacy (e.g., Greenleaf et al, 2011; O'Reilly & McNamara, 2007).

Former research produced inconclusive results regarding relations of verbal skills to content knowledge und scientific inquiry in science and biology. On the one hand, verbal skills could be more important for content knowledge since students have to understand a specific terminology and reading in the science classroom is often accompanied by the learning of factual knowledge (Greenleaf et al, 2011). On the other hand, science inquiry (e.g., the process aspects of science literacy) is often conveyed through verbal skills since students have to read and write scientific explanations to understand and formulate hypotheses (Greenleaf et al, 2011).

Research has revealed a high correlation between scientific literacy and general cognitive abilities as well as verbal skills. In this study, we connect these constructs with the two subdimensions of biology literacy—content knowledge and scientific inquiry—to study their impact in more detail.

## HYPOTHESES

There exists broad consensus in the literature that biology literacy consists of separable subdimensions that can be measured in LSA. Research validating such an assumption, however, is rare. Therefore, we used a large pool of biology items that was specifically designed for the national assessment of the German Educational Standards at the end of lower secondary school and allows for testing the multidimensionality of biology literacy. We were interested in the underlying structure of the item pool (number of separable subdimensions) and how different subdimensions of biology literacy are related to general cognitive abilities and verbal skills. Bearing this in mind, we derived the following predictions:

Hypothesis 1a.: Content knowledge and scientific inquiry form two empirically distinguishable subdimensions of biology literacy.
Hypothesis 1b.: These two subdimensions of biology literacy are highly correlated.
Hypothesis 2.: General cognitive abilities and verbal skills substantially predict content knowledge and scientific inquiry in biology.

For the first two hypotheses, we analyze the two subdimensions of biology literacy by applying competing Conformative Factor Analysis (CFA) models.

Previous research on the dimensionality of scientific literacy led us to develop two additional hypotheses that focus on the differential effects of relevant covariates on both subdimensions of biology literacy. The corresponding results could provide further insights into similarities and differences of biology literacy and related abilities.

Hypothesis 3.: Content knowledge in biology is stronger related to verbal skills than to general cognitive abilities.
Hypothesis 4.: Scientific inquiry in biology is stronger related to general cognitive abilities than to verbal skills.

To scrutinize the third and fourth hypothesis, we regress the two factors of biology literacy on general cognitive abilities and verbal skills as predictors.

## METHODS

### Participants

We used a data set from a large pilot study, which was carried out by the German Institute for Educational Quality Improvement (IQB) to analyze psychometric properties of items that were developed to assess educational outcomes described in the German Educational Standards for biology at the end of lower secondary school. Since the standards for lower secondary level aim at the outcome right before graduation (or before continuing on to upper secondary level), we investigated 10th graders. We used a subsample of 3,165 students who worked on booklets containing biology items with an average of 11 students per class (see section Design and Procedure). Of the participating students, 22.7% had a migration background. The students came from academic track schools (47%, preparing students for university), intermediate track or mixed track schools (53%, both school types prepare most students for vocational education). Half of the sample was female (51%), and the mean age was 15.50 years (*SD* = 2.16).

### Design and Procedure

The study took place in autumn 2009 in eight German federal states. The sample consisted of students who planned to finish school with a 10th‐grade degree or higher. The overall sample was derived as follows: Eight out of sixteen federal states (North Rhine‐Westphalia, Schleswig‐Holstein, Berlin, Saxony, Bavaria, Baden‐Wurttemberg, Hesse, and Thuringia) were chosen that reasonably represent the heterogeneity of the federal educational system in Germany. For each state, 20 schools were randomly selected. Finally, two complete classes of 10th graders were randomly selected in each school. This procedure resulted in a sample of 299 classes from 158 schools. Seventeen schools only provided one 10th grade.

In each class, booklets containing tasks in biology, chemistry, or physics were administered (see section Measures design and instruments). To ensure the objectivity of the assessment, trained test administrators strictly followed a fixed procedure. Testing time was 3½ hours. After the science achievement part (two 60 minutes blocks with a 15 minutes break) participants worked on a measure on general cognitive abilities and an indicator of verbal skills for 20 minutes (after a 15 minutes break). Finally, students worked for 40 minutes on a questionnaire asking for the family background, motivational aspects, and learning opportunities in school.

The biology booklets contained items assessing outcomes defined in the German Educational Standards. Similar to other LSAs, the booklets were administered as paper‐and‐ pencil tests. Such tests usually have some limitations when assessing scientific inquiry. In contrast, hands‐on tests that are often regarded as better measures for scientific inquiry have repeatedly been shown to be insufficiently reliable (Solano‐Flores & Shavelson, 1997) and are rarely applied in LSAs due to their high costs.

To keep the individual workload within acceptable limits, an incomplete block design (IBD) was applied to the science achievement test (Frey, Hartig, & Rupp, 2009). Each student only received a subsample of the total item pool consisting of 270 items. An introductory text (stem) was followed by one to seven questions (on average three questions) with either a multiple‐choice format (MC) or an open response format. Open response items with short constructed response formats required students to answer with a few words or a sentence, whereas items with an extended constructed response format required participants to write a short paragraph. The different item formats were distributed equally across the two subdimensions. About 50% of the items were MC items. The remaining half of the items either had an open response or a short‐constructed response format.

### Measures

The measures on scientific literacy stem from the German Educational Standards item pool and underwent an item selection. Items with extreme difficulties (*b* < –3.00 or *b* > 3.00), low item‐total correlations (*r* < .25) and substantial infit values according to the Rasch model (infit weighted mean square [WMNSQ] > 1.15) were excluded and not part of the pool which was used for our analyses.

#### Content Knowledge Test

**Figure 1:**
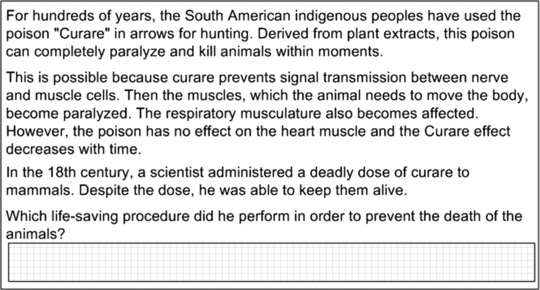
Example item for content knowledge (Curare).

Content knowledge in biology was assessed by means of 117 items. Students had to answer questions related to the following basic concepts: (a) evolution (29 items), (b) structure and function (51 items), and (c) system (37 items). An example item is given in Figure 1: After an introductory text describing the influence of the poison Curare on different animal muscle types, students had to indicate which type of life‐saving action is most effective. The example deals with the concept structure and function, so students had to apply their content knowledge to correctly answer the question. The model‐based reliability of the scale for biology content knowledge was McDonald's ω = 0.98 (McDonald, 1999).

#### Scientific Inquiry Test

**Figure 2:**
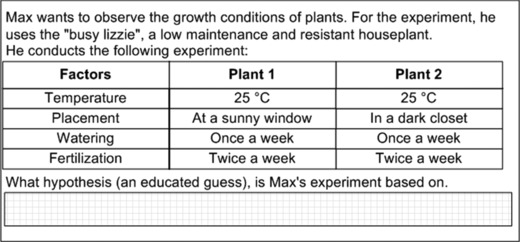
Example item for scientific inquiry (Busy Lizzie).

A total of 153 items covered scientific inquiry in biology. The underlying idea of these items relates to the scientific process, which means that students needed to handle and apply their knowledge to process aspects of biology literacy. To solve such items, students had to answer questions related to (a) scientific investigation (105 items), to (b) scientific models (25 items) or to (c) the scientific theorizing (22 items). For example, after an introductory text explaining an experiment on the growing conditions of plants, students had to formulate the hypothesis leading to the experiment (see Figure 2). The model‐based reliability of the scale for biology scientific inquiry was McDonald's ω = 0.96.

#### Verbal Skills

Two cloze tests were used as global indicators of verbal skills in German (mother tongue for most students). Students had to fill in 20 blanks per text to complete sentences in a meaningful manner. Only grammatically and orthographically flawless answers were coded as correct. Owing to insufficient psychometric properties, we decided to exclude several items from further analyses. The selection process followed a two‐step procedure: First, items with extreme item difficulty were excluded (*p* < .05 or *p* > .95). Second, items leading to considerable misfit in a unidimensional measurement model were eliminated. Item misfit was examined by stepwise exploratory factor analysis (Kano & Harada, 2000). For the first cloze test—a text book passage about dream jobs—six items had to be eliminated resulting in sufficiently good model fit values (root mean square of approximation [RMSEA] = 0.04, comparative fit index [CFI] = 0.93, Tucker Lewis index [TLI] = 0.92). For the second cloze test—a text from the book *Life of Pi*—another six items had to be excluded, obtaining good model fit (RMSEA = 0.06, CFI = 0.95, TLI = 0.94). The model‐based reliability of the two cloze tests were McDonald's ω_CT1_ = 0.89 and McDonald's ω_CT2_ = 0.90.

#### General Cognitive Abilities

A nonverbal scale with 30 MC‐items from a German test on cognitive abilities was administered (subtest N2 from the Kognitiver Fähigkeitstest [Cognitive ability test]; Heller & Perleth, 2000). More precisely, students were shown a pair of geometrical figures, for instance, a big dark circle and a small white circle. After being shown a third geometrical figure (a big dark rectangle) students had to induce the underlying rule and find the correct solution among five response alternatives. All items were represented by a common underlying factor (RMSEA = 0.03, CFI = 0.96, TLI = 0.96; McDonald's ω_CT2_ = 0.93).

### Statistical Analyses

Since each student only received a subset of the items (see section Design and Procedure), we could not compute sum scores but needed to apply probabilistic test theory (also called item response theory [IRT] or latent trait theory). Through test designs which link items between students, the probabilistic test theory allows for simultaneous estimation of all items for each student even though they have only answered a subset of them. Additionally, within the probabilistic theory unobservable constructs or traits—like mathematical, scientific or biology literacy—are modeled as latent factors. These factors can be described by several manifest (observable) indicators, which in our case are the individual test items. Since this modeling approach takes into account measurement errors of the manifest indicators, the latent factors are also uncontaminated by measurement errors (Wang & Wang, 2012). Therefore, we applied models within the probabilistic frame. All statistical analyses were carried out with Mplus 6.1 (Muthén & Muthén, 1998–2010). All manifest variables were dichotomously scored (0 = wrong answer, 1 = right answer). We used the relative fit indices Bayesian Information Criterion (BIC), the Akaike's Information Criterion (AIC), and the consistent Akaike's Information Criterion (cAIC) to check whether a one parameter logistic (1PL, factor loadings are restricted to be equal) or a two parameter logistic (2PL, factor loadings vary) model fits the data better for each construct (see Table A1 in the Appendix). This measurement model check was necessary before building the structural models. Note, however, that IRT models are directly convertible into classical test theory, i.e. our latent 1PL‐ and 2PL‐models correspond to factor analytical models with fixed (1PL) and freely (2PL) estimated factor loadings. Estimation of all models was based on the robust maximum likelihood (Kline, 2011). Owing to our incomplete block design, Mplus does not provide χ²‐statistics. Accordingly, RMSEA and CFI/TLI were not reported for these models.

**Figure 3:**
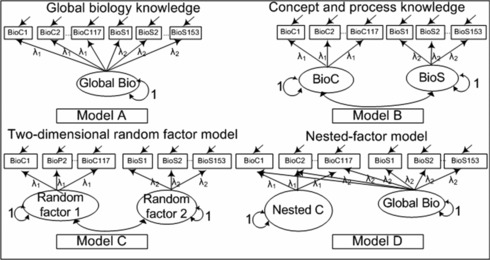
Competing measurement models of abilities in biology. BioC = Biology content knowledge, BioS = scientific inquiry.

For content knowledge and scientific inquiry in biology, the 1PL measurement models fitted the data better regarding at least two of the relative fit indices. For the covariates, the 2PL measurement models provided best fit to the data regarding at least two indices. Accordingly, these two different types of measurement models were used in the CFAs and SEMs. For the first two hypotheses, four competing models were estimated (see Figure 3) and compared according to model fit as well as in terms of the Satorra–Bentler adjusted χ²‐difference test and the Wald test (Bollen, 1989). The Satorra–Bentler adjusted χ²‐difference test compares the overall fit of two nested models and was performed on the basis of the log likelihood. The Wald test compares two models that differ in one specific parameter which is constrained in one of the models. Both tests were available even though the χ²‐statistics were not provided.

The first model was a one‐dimensional model. The second model was a theory‐driven two‐dimensional model, in which 117 items loaded on the latent factor content knowledge and the remaining 153 items on the latent factor scientific inquiry. The theory‐driven two‐dimensional model with two latent factors—content knowledge and scientific inquiry—should represent the data better than a one‐dimensional model. In the third model, the items were randomly assigned to two arbitrary latent factors with 117 and 153 items. This model with any two dimensions that are not theory driven should also fit poorer to the data than the theory‐based two‐dimensional model. The fourth and final model was a nested factor model in which all items were assigned to a general biology factor, and additionally the 117 items of content knowledge were loading on a nested factor which was uncorrelated with the general factor. If this model represents the data better than the theory‐driven two‐dimensional model, we cannot view biology literacy as a two‐dimensional model but rather as one global literacy and a separate knowledge dimension. Because 1PL measurement models described the data more accurately than 2PL models, the factor loadings for each factor were fixed, meaning that they were the same within each dimension.

The data yielded two main challenges. First, the sampling procedure led to nested data (students nested within classes, classes nested within schools). In the literature on hierarchical data (e.g., Hox, 2010), it is argued that due to strong intraclass correlations of achievement measures (e.g., variance in outcome measures explained by class‐ or school‐membership) the effective sample size of class‐based samples is much lower than the number of real participants in the study. This typically leads to an underestimation of standard errors and consequently to an inflation of type‐I errors (rejection of true null hypotheses) resulting in spurious significant results. In our study, the intraclass correlations of the achievement measures were above .30. These values can be read as a reliability value of a student's individual value to the average class’ or school's value. Therefore, we decided to account for the hierarchical data structure by using the Mplus option TYPE = COMPLEX that is designed to estimate unbiased standard errors in clustered samples. Second, owing to the IBD the variance–covariance coverage was incomplete. Since the booklets were assigned randomly to students, missing values can be categorized as missing completely at random. Mplus offers the full information maximum likelihood procedure for these kinds of unsystematic failures. This model‐based approach estimates the parameters and standard errors on the basis of the observed values (Enders, 2010).

## RESULTS

The first two hypotheses addressed the dimensionality of biology literacy with respect to content knowledge and scientific inquiry. Table 2 shows fit indices of the four models presented above: the one‐dimensional model, the theory‐driven two‐dimensional model, the two‐dimensional model with random assignment of items to factors, and the nested factor model.

**Table 2:** Model Fit of Four Competing Measurement Models on Biology Literacy

Model		Final Deviance	AIC	cAIC	BIC	Parameter	Deviance Change	χ²‐Diff. Test	Wald Test
A	One‐dimensional (global biology knowledge)	70,513	71,057	72,977	72,705	272			
B	Two‐dimensional (content and scientific inquiry)	70,488	71,034	72,962	72,689	273	–25	.00^a^, [Link]	19.55 (1), .00^b^, [Link]
C	Random two dimensional	70,555	71,101	73,028	72,755	273	42		0.74 (1), .39^b^, [Link]
D	Nested factor	70,493	71,037	72,957	72,685	272	–20		

*Note*. AIC = Akaike Information Criterion, cAIC = consistent Akaike Information Criterion, BIC = Bayesian Information Criterion.

a *p* value.

b Values represent χ² values (*df*) and *p* values.

³Comparison of Model B/Model C to Model A. The Wald test was only applied to test whether the correlation between the latent factors was different from 1; reported values are as follows: Wald test statistic, (*df*), *p* value

The theory‐driven two‐dimensional model and the nested factor model fitted the data quite well. The final deviance was lowest for the theory‐driven two‐dimensional model (model B), that is, 25 points below the more parsimonious model with a single biology factor (model A). Compared to this baseline model, the fit of the random factors model (model C) deteriorated considerably by 42. The AIC of model B and model D were almost similar and the lowest (improvement of 23 for model B and 20 for model D, compared to model A). The same held true for the BIC, but here the fit of model D improved by 20 and of model B by 16 compared to model A. Comparing the cAIC for the same models shows that model D improves by 20 and model B by 15.

Evaluating these results—bearing in mind that we were interested in the correlation between the two subdimensions of biology literacy—we favored model B. To further clarify the model comparison, we applied two model tests: The Wald test and Satorra–Bentler adjusted χ²‐difference test. The χ²‐difference test can only be applied to model B because solely this model is nested in model A. The Satorra–Bentler adjusted χ²‐difference test comparing models A and B was significant, indicating that model B fitted the data significantly better than model A.

The two latent factors of this model B, content knowledge and scientific inquiry, were highly correlated with ρ = .89 (*SE* = 0.03). In comparison, the correlation between two random factors amounted to ρ = .99 (*SE* = 0.02). The Wald test was used to test the correlation of models B and C against a value of one. The test was statistically significant for model B, indicating that the correlation between the two factors was statistically different from unity. The same test was not significant for model C, that is the correlation between the two random factors did not differ significantly from unity. The results show that content knowledge and scientific inquiry were two empirically separable subdimensions of biology literacy but highly correlated.

**Figure 4:**
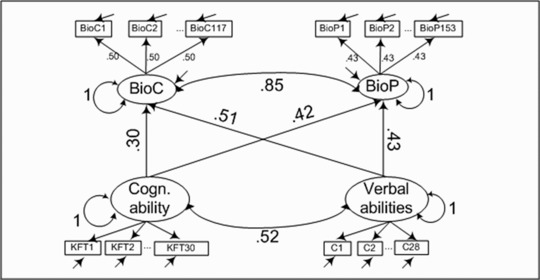
The SEM incorporating the theory‐driven two‐dimensional model of biology and the covariates verbal abilities and general cognitive ability.

Concerning hypothesis two to four, we assumed that general cognitive abilities and verbal skills would predict interindividual differences in content knowledge and scientific inquiry to a certain degree. Figure 4 depicts the findings of the corresponding SEM. Both latent factors of biology literacy were strongly related to the covariates. General cognitive abilities and verbal skills explained 51% of interindividual differences in content knowledge and 55% in scientific inquiry. We thus found support for the assumption that general cognitive abilities and verbal skills predicted biology literacy to a certain degree, but that there is still variance not explained by these two predictors.

To answer the last two hypotheses, we compared the regression coefficients expressing the effects of general cognitive abilities and verbal skills on content knowledge and scientific inquiry (see Figure 4). Differential effects could be found for content knowledge, that is, verbal skills were more predictive (β_language_ = .51 vs. β_cog. abilities_ = .30). In contrast, scientific inquiry was equally affected by both constructs (β_language_ = .43 vs. β_cog. abilities_ = .42). The correlation between both predictors amounted to ρ = .52.

We also applied the Wald test to the two regression coefficients on content knowledge by checking for equality of the two regression coefficients. The two coefficients differed significantly (χ² = 19.86; *df* = 1; *p* < .001). For scientific inquiry, no statistically significant difference between the two regression coefficients could be found (χ² = 0.10; *df* = 1; *p* = .76). Thus, for content knowledge we found a differential picture as assumed by the third hypothesis. Verbal skills were stronger related to content knowledge than general cognitive abilities to the same dimension. Such a differentiated pattern could not be found for the subdimension of scientific inquiry (Hypothesis 4). The fact that differential predictive effects for verbal skills were found supports the empirical and theoretical separation of the two subdimensions.

## DISCUSSION

Although from a conceptual perspective numerous authors advocate the existence of different subdimensions of scientific literacy such as content knowledge and scientific inquiry, there are only few studies investigating the internal structure of measures of scientific literacy empirically. Notwithstanding this research gap, LSAs all around the world assessing scientific literacy are used at the moment as a tool to monitor educational systems nationally and internationally. Most of the frameworks that form the basis for LSA rely on an a priori postulated internal structure by political stakeholders and science educators. In this vein, the German Educational Standards in biology define outcomes for different subdimensions of biology literacy (KMK, 2005a). The aim of educational assessment to track such outcomes according to these dimensions raises questions on the validity of the measures. First, a test that is designed to assess content knowledge and scientific inquiry needs to be empirically validated with respect to its theoretically derived structure. Second, the relations of a test that is used to measure knowledge, skills, or abilities in a certain domain—in our case biology—to covariates have to be analyzed. Both issues have been addressed in this article. By comparing four competing models, we found evidence for a theory‐driven two‐dimensional model. The two subdimensions—content knowledge and scientific inquiry—correlated highly but below one. This finding adds empirical evidence to previous research (e.g., Senkbeil et al., 2005).

In a lot of curricular and educational materials of science educators content knowledge and scientific inquiry is already integrated. Our findings support this conception and thus the work of science educators around the world. Based on our data, we argue that both subdimensions should be included in any LSA framework for biology literacy. Countries like Germany that are still on their way to meaningfully incorporate scientific inquiry in their documents for the educational assessment system also get empirical evidence supporting their strategy. To make LSA reporting even more meaningful, future research should also explore the internal structure of biology literacy at a more fine‐grained level. For example, results on the facet level of scientific inquiry would give stakeholders and curriculum developers more detailed information for specific challenges that need to be targeted.

The high correlation between the two subdimensions allows for at least two interpretations. First, students could activate both subdimensions while solving scientific problems. Second, a certain part of the high correlation between content knowledge and scientific inquiry will stem from a common hierarchical factor that we could call “biology literacy g”. Further in‐depth hierarchical nested‐factor analyses could separate these factors.

Both subdimensions of biology literacy were substantially related to general cognitive abilities and verbal skills, accounting for half of the variance in each subdimension. This finding supports previous studies showing that these two abilities are strong predictors for interindividual differences in academic achievement (Baumert et al., 2009; Gustafsson & Balke, 1993; Rolfhus & Ackerman, 1999). We have to point out that we did not expect a perfect explanation of biology literacy by these two covariates since there should at least be a domain‐specific component that can, for example, be attributed to differing learning opportunities in biology classes and in out‐of‐school experiences. We also demonstrated differential predictive patterns for the two subdimensions. While both predictors contributed equally to scientific inquiry, verbal skills were stronger related to content knowledge. Stronger than general cognitive abilities, verbal skills seem to play a major part in knowledge acquisition in science (e.g.; Brown et al., 2005; Ford et al., 2006; Taboada & Rotherford, 2011).

## LIMITATIONS AND FUTURE RESEARCH

To correctly cover literacy in a specific domain, any assessment of students’ achievement has to mirror the underlying structure. Our results suggest that different subdimensions of biology literacy do exist and should be covered in LSA. However, answers to the question how to foster learning within the two subdimensions cannot be derived from the present study. Students proficient in one dimension might benefit from knowledge in the other dimension, for example, students may benefit from knowledge about weather phenomena when learning how to interpret a table on precipitation or they benefit from being able to read the table with regard to acquiring knowledge about weather phenomena. Less proficient students are presumably not competent in transferring knowledge from one subdimension to the other.

As another limitation, our study provided no insights on how the correlation of the two subdimensions changes over time when learning science. Nevertheless, it can offer insights into the structure of biology literacy at a specific and decisive time point, in our study at the end of lower secondary school. We could show that the structural pattern of biology literacy consists of a content knowledge and scientific inquiry dimension and that these two are highly related. An interesting question is why—bearing in mind the conceptual differences—the two subdimensions of biology correlate so highly. One reason could be that by learning within one subdimension, students seem to gain knowledge and skills in the other subdimension as well, that is, the domains are mutually dependent. How skills and abilities in one subdimension promote learning in the other domain is, however, subject to future research. A longitudinal study would offer the possibility to investigate the processes of acquisition for both dimensions and their interrelation throughout educational learning.

To transfer our results into the school context, researchers should perform quasi‐experimental studies incorporating both dimensions of biology literacy. These studies should, for instance, involve teaching scientific inquiry (e.g., coming up with hypotheses, drawing up an own experiment, finding different interpretations for experimental data) and investigate its impact on content knowledge. Once these mechanisms are revealed, the interplay of the two subdimensions could be an integral part of the sciences curricula. Until now, the science curricula in Germany incorporate both subdimensions, but neglect their interplay (e.g., Senatsverwaltung für Bildung, Jugend und Sport [SBJS], 2006).

By investigating the effects of covariates, we showed that general cognitive abilities and verbal skills are important determinants of content knowledge and scientific inquiry in biology, both explaining 51% of the variance of content knowledge and 55% in the case of scientific inquiry. They are significant sources of performance differences in biology literacy that should be acknowledged in learning and teaching.

The present study has some additional limitations: We assessed only two subdimensions of biology literacy—content knowledge and scientific inquiry. However, biology and scientific literacy are composed of more process dimensions such as argumentation skills socioscientific decision making or epistemological beliefs (Gräber et al., 2002; Hodson, 1992; Osborne, 2007). Nevertheless, independent of the number of proposed subdimensions research has to show that such subdimensions can be distinguished empirically. We conducted such analyses with two widely agreed on subdimensions of biology literacy and found some evidence for their distinctiveness, although the correlation of .89 was quite high. The example items in Figures 1 and 2 might suggest that the two dimensions could be caused by differences between the item types rather than by the different contents (knowledge vs. inquiry). We thus recommend conducting future studies in which items of both dimensions are based upon the same item stem to dispel these doubts. Furthermore, incorporating all aspects of biology literacy would draw a more comprehensive picture.

Another limitation is that LSAs with such a large sample size cannot reveal the student's in‐depth level of understanding. Analyses on this topic need different designs and methods in smaller groups and targets, for instance, the development within facets of scientific inquiry in science or biology. Examples of these in‐depth analyses already exist (Kremer et al., 2014).

Furthermore, we collected data on the process aspect of biology literacy by means of a paper‐pencil test. Such a format can only cover selected skills but not the whole construct. The hands‐on skills in scientific situations could be approximated more closely using a performance test. However, such tests are rarely applied in LSAs due to reliability issues (Solano‐Flores & Shavelson, 1997) and high costs. A research desideratum of future studies is thus to combine different modes of assessment covering different aspects of scientific literacy.

## CONCLUSION

The present study showed that biology literacy as assessed in typical LSAs does comprise distinct, but highly related subdimensions that show differential associations with covariates. However, more research has to be done to (a) grasp the internal structure of biology and scientific literacy, (b) establish the crucial cognitive and motivational determinants for learning within these subdimensions, and (c) gain a better understanding of the developmental processes underlying the formation of both dimensions. Furthermore, the development of content knowledge and scientific inquiry as well as the influence of general cognitive abilities and verbal skills on learning in science remains to be investigated in longitudinal studies. Finally, we strongly recommend that future studies in the field of LSA should put more emphasis on the validation of the assessment instruments, before they are used for policy decisions.

## Appendix

**Table A1:** Goodness‐of‐Fit (BIC) of Two Different Probabilistic Models (1PL vs. 2PL for the Achievement Measures in the Present Study)

Construct	Model	BIC	AIC	cAIC
Ck	1PL	30,212.62	29,507.22	30,330.62
	2PL	30,905.99	29,507.15	31,139.98
Si	1PL	43,139.02	42,214.19	43,293.03
	2PL	43,971.50	42,133.86	44,277.50
Vs	1PL	77,683.33	77,507.60	77.712,34
	2PL	77,033.14	76,693.79	77.089,13
Gca	1PL	96,540.95	96,353.10	96.000,53
	2PL	95,940.52	95,576.93	96.571,96

*Note*. BIC = Bayesian Information Criterion, AIC = Akaike's Information Criterion, consistent Akaike's Information Criterion = cAIC, 1PL = one parameter logistic, 2PL = two parameter logistic, ck = content knowledge, si = scientific inquiry, vs = verbal skills, gca = general cognitive abilities.
